# Delayed Diagnosis of a Subungual Glomus Tumor of the Right Ring Finger Successfully Treated With Nail Apparatus Preservation

**DOI:** 10.7759/cureus.109718

**Published:** 2026-05-27

**Authors:** Ahmed Alsaedawy A Albeshlawy, Hazem A Osman

**Affiliations:** 1 Orthopaedic Surgery, Khafji General Hospital, Khafji, SAU

**Keywords:** case report, delayed diagnosis, finger pain, glomus tumor, hand surgery, nail apparatus, nail preservation, neoplasm, subungual tumor, transungual approach

## Abstract

Subungual glomus tumors are rare benign vascular neoplasms arising from the neuromyoarterial glomus body. Despite a characteristic clinical triad of severe localized pain, point tenderness, and cold hypersensitivity, prolonged diagnostic delay is common and represents a significant source of patient morbidity. A 39-year-old male presented with a 10-year history of severe subungual pain in the right ring finger that remained undiagnosed across multiple consultations with different specialists. Plain radiography excluded bony involvement, and MRI confirmed a well-defined T2-hyperintense subungual lesion consistent with a glomus tumor. Complete excision was performed via a transungual approach with full nail apparatus preservation. Histopathology confirmed a benign glomus tumor (glomangioma) with diffuse smooth muscle actin positivity. The patient was pain-free at the six-month follow-up with no recurrence. This case underscores the importance of clinical vigilance in patients with chronic unexplained fingertip pain. Early application of the Love and Hildreth tests, followed by MRI, enables timely diagnosis. Complete surgical excision with nail preservation achieves excellent functional and cosmetic outcomes and prevents years of unnecessary patient suffering.

## Introduction

Glomus tumors are rare benign neoplasms originating from the neuromyoarterial glomus body, a specialized thermoregulatory arteriovenous shunt composed of modified smooth muscle cells (pericytes), afferent arterioles, and efferent collecting venules [1]. They account for approximately 1-5% of all soft tissue tumors of the hand, with the subungual region of the fingers representing the most common anatomical site [2]. The glomus body is particularly dense in the fingertip subungual region, explaining the predilection of these tumors for this location [3].

The classic diagnostic triad of severe lancinating pain, exquisite point tenderness, and cold hypersensitivity is highly specific for subungual glomus tumors and has been recognized since their original description by Masson in 1924. Despite this characteristic presentation, diagnostic delay of 7-10 years is consistently documented across published series [2,4]. Contributing factors include the tumor's small size (typically 2-10 mm), its concealed subungual location, and the disproportionate severity of pain relative to the paucity of objective findings, which frequently misleads non-specialist clinicians toward alternative diagnoses such as paronychia, nail dystrophy, peripheral neuropathy, or complex regional pain syndrome [5].

Two highly sensitive bedside clinical tests are available to support diagnosis: the Love test, in which focal application of pressure with a blunt pin over the lesion precisely reproduces the characteristic pain [6], and the Hildreth test, in which application of a digital tourniquet abolishes pain, which returns upon tourniquet release, confirming a vascular etiology [7]. MRI has emerged as the gold standard imaging investigation, offering sensitivity of 90% and a positive predictive value of 97%, and is essential for accurate preoperative localization and surgical planning [3]. Complete surgical excision is curative, with recurrence rates of 4-15% primarily attributable to incomplete removal [5].

Surgical management of subungual glomus tumors has evolved considerably over recent decades. The transungual approach, first popularized by Van Geertruyden et al. [1], remains the most widely employed technique for centrally located lesions, offering direct visualization of the tumor bed and facilitating complete excision. Alternative approaches including the lateral subperiosteal and periungual routes have been described, each with specific indications based on the tumor location [8-10]. Regardless of approach, complete excision is the paramount surgical objective; recurrence rates of 4-15% are predominantly attributable to incomplete removal rather than surgical technique [5]. Nail apparatus preservation, achieved by replacing the elevated native nail plate as a biological dressing, has been shown to promote superior cosmetic regrowth and minimize postoperative nail bed hypersensitivity [5,10]. Glomangioma, the histopathological subtype most commonly encountered, is characterized by a predominance of vascular channels relative to glomus cells, with diffuse smooth muscle actin (SMA) positivity and variable CD34 expression on immunohistochemistry [1,4]. Confirmation of the diagnosis by histopathological examination with immunohistochemical profiling is essential in all cases. We present a case that illustrates the substantial patient burden imposed by prolonged diagnostic delay and demonstrate that a transungual approach with full nail apparatus preservation achieves excellent long-term outcomes. This work has not been previously presented at any conference or scientific meeting.

## Case presentation

Patient history and presentation

A 39-year-old right-hand-dominant male presented to our orthopedic outpatient clinic with a 10-year history of severe, intermittent, lancinating pain localized to the subungual region of the right ring finger. The pain was markedly exacerbated by cold exposure and minor contact, significantly impairing occupational performance and daily activities. The patient had attended multiple hospitals over the preceding decade, receiving evaluations from general practitioners, internists, and dermatologists. Previous working diagnoses had included paronychia, nail dystrophy, and peripheral neuropathy; no definitive cause had been identified, and symptomatic treatments had provided no sustained relief.

Physical examination

Clinical examination revealed faint bluish discoloration beneath the nail plate of the right ring finger. The Love test (point pressure with a blunt pin over the suspected lesion) was strongly positive, precisely reproducing the characteristic lancinating pain [6]. The Hildreth test (application of a digital tourniquet abolishing pain, with pain reproduction upon tourniquet release) was also positive, confirming a vascular etiology [7]. Cold sensitivity was confirmed by application of an ice pack to the fingertip.

Imaging

**Figure 1:**
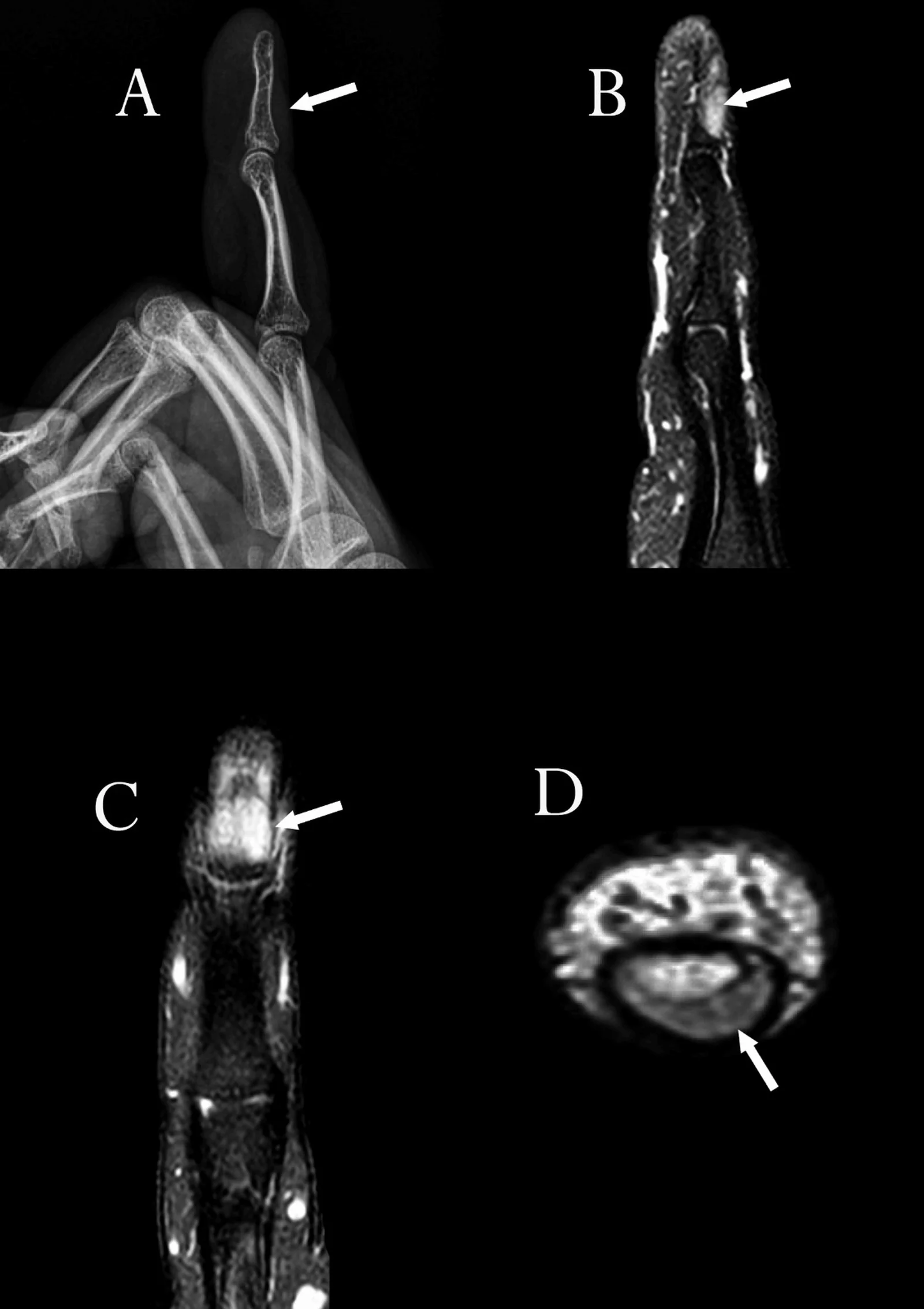
(A) Plain radiograph of the right hand demonstrating no bony erosion, periosteal reaction, or calcification at the distal phalanx of the ring finger, effectively excluding bony pathology. (B) Sagittal T2-weighted MRI demonstrating a well-defined hyperintense subungual lesion at the distal phalanx of the ring finger. (C) Coronal T2-weighted MRI showing the hyperintense subungual lesion. (D) Axial MRI cross-section confirming the subungual location of the lesion and delineating its relationship to adjacent structures. No cortical erosion is identified on any sequence.

Plain radiograph of the right hand was performed as the initial imaging investigation and demonstrated no bony erosion, periosteal reaction, or calcification at the distal phalanx of the right ring finger (Figure 1A). Although plain radiography is typically unremarkable in glomus tumors, it is valuable in excluding cortical erosion, bony involvement, or alternative diagnoses such as enchondroma or osteoid osteoma, and represents an essential first-line investigation. MRI of the right ring finger was subsequently performed and demonstrated a well-defined, T1-hypointense, T2-hyperintense ovoid lesion measuring approximately 9 mm in the subungual region of the distal phalanx, with homogeneous gadolinium enhancement consistent with a glomus tumor. No cortical erosion or adjacent bony involvement was identified (Figures 1B-1D).

Surgical findings and technique

**Figure 2:**
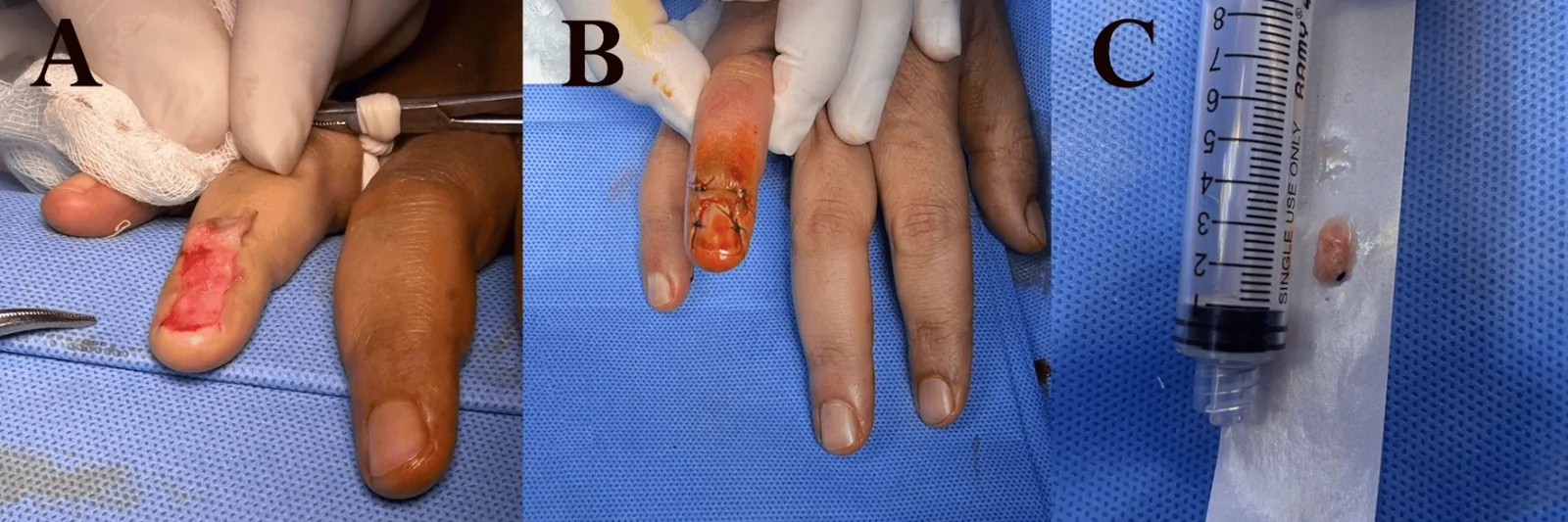
(A) Intraoperative photograph demonstrating the exposed subungual nail bed following nail plate elevation, with the reddish-pink glomus tumor nodule directly visualized. (B) Postoperative appearance showing the native nail plate repositioned and secured with interrupted sutures as a biological dressing. (C) Gross specimen of the excised glomus tumor placed adjacent to a ruler for size reference, measuring approximately 10 mm.

After informed consent was obtained, surgery was performed under digital ring block anesthesia with a finger tourniquet to provide a bloodless operative field. The nail plate was carefully elevated from the nail bed, preserving its structural integrity. The subungual lesion was directly visualized as a reddish-pink, well-circumscribed, firm nodule (Figure 2A). Complete excision was performed with clear surgical margins. The nail bed was repaired with interrupted 6-0 absorbable sutures and the native nail plate was replaced and secured as a biological dressing (Figure 2B). The excised specimen measured approximately 10 mm (Figure 2C).

Pathological findings

Gross examination of the specimen received at the Dammam Regional Laboratory (Eastern Province, Saudi Arabia; CAP-accredited) revealed a whitish soft tissue fragment measuring 0.7 × 0.7 × 0.3 cm (Figure 2C). Microscopic examination demonstrated solid and syncytial proliferation of round cells with bland nuclei surrounding scattered blood vessels, with associated smooth muscle cells, no mitotic activity, no necrosis, and no nuclear atypia. Focal stromal hyalinization was noted. Immunohistochemically, glomus cells showed diffuse positivity for SMA and focal positivity for CD34, confirming the diagnosis of a benign glomus tumor (glomangioma). 

Postoperative course and follow-up

At the six-month postoperative follow-up, the patient reported complete and sustained resolution of pain and cold sensitivity from the first postoperative week. Clinical examination confirmed a full range of motion, no tenderness, and satisfactory nail regrowth with no evidence of local recurrence.

## Discussion

This case exemplifies the prolonged diagnostic odyssey that is characteristic of subungual glomus tumors. A delay exceeding 10 years, as seen in our patient, is not exceptional; published series document average delays ranging from 4 to 13 years [2,4]. The combination of the tumor's small size, its concealment beneath the nail plate, and the disproportionate severity of pain relative to physical findings routinely leads to misdiagnosis. The prior misattribution of symptoms to paronychia and peripheral neuropathy in our patient reflects the most commonly reported diagnostic errors in this condition, as documented by Bhaskaranand and Navadgi [8] and confirmed in multiple subsequent series.

The Love test and Hildreth test are simple, highly sensitive bedside tools that should be applied routinely in patients with chronic unexplained fingertip pain [6,7]. The Love test has a reported sensitivity of 96% and specificity approaching 100%, while the Hildreth test demonstrates a sensitivity of 77% and specificity of 100% [6,7]. In this case, neither test had been performed during any prior consultation, a failure that prolonged the patient's suffering by years. Wider education of non-specialist clinicians regarding these simple tests has direct potential to reduce diagnostic delay.

MRI is the imaging investigation of choice, providing excellent soft tissue contrast, precise lesion localization, and assessment of bony involvement [3]. The T1-hypointense, T2-hyperintense signal with homogeneous gadolinium enhancement, as demonstrated in our case, is highly characteristic of a glomus tumor. Al-Qattan et al. reported MRI sensitivity of 90% and a positive predictive value of 97% for subungual glomus tumors, supporting its role as the definitive preoperative investigation [9]. Ultrasound represents an accessible alternative but has inferior sensitivity for lesions smaller than 5 mm and is more operator-dependent [3].

The transungual approach provides optimal direct visualization of centrally located subungual lesions and is the most widely reported surgical technique [5]. Nail apparatus preservation is increasingly prioritized as a key surgical objective. Replacement of the native nail plate as a biological dressing promotes superior cosmetic nail regrowth and minimizes postoperative nail bed hypersensitivity compared with leaving the nail bed exposed [5,10]. Recurrence rates of 4-15% are predominantly associated with incomplete tumor removal rather than the surgical approach employed [5]. The complete symptom resolution and absence of recurrence at six-month follow-up in our patient are consistent with outcomes reported in comparable published series [2,4,5].

The histopathological subtype confirmed in our case, glomangioma, is characterized by a predominance of vascular channels relative to glomus cells, with diffuse SMA positivity and variable CD34 expression on immunohistochemistry, as observed in our specimen [1,4]. This subtype is the most common variant of glomus tumor and carries the same favorable prognosis as the classic form when completely excised. The absence of mitotic activity, necrosis, and nuclear atypia confirmed the benign nature of the lesion. The clinical and pathological features of our case are consistent with those reported across published series. Van Geertruyden et al. reported an average symptom duration of 10 years before treatment in a series of 51 patients, reflecting the diagnostic delay observed in our case [1]. Morey et al. similarly documented delays of 4 to 13 years and attributed this pattern to the disproportionate severity of pain relative to objective clinical findings [2]. The favorable outcome achieved in our patient, complete pain resolution from the first postoperative week and absence of recurrence at six months, is consistent with published recurrence rates of 4-15% and corroborates the efficacy of the transungual nail-preserving approach when complete excision is achieved [5,10]. From a public health perspective, this case reinforces the need for systematic incorporation of the Love and Hildreth tests into the physical examination of any patient presenting with chronic unexplained fingertip pain, regardless of prior diagnoses or treating physician's specialty. The failure to apply these simple, high-yield bedside tests across multiple consultations over 10 years represents a preventable gap in clinical practice that is well-documented in the literature and deserves targeted educational intervention [2,8].

## Conclusions

Subungual glomus tumors should be considered in the differential diagnosis of any patient presenting with chronic unilateral fingertip pain and cold hypersensitivity, regardless of the duration of symptoms or the number of prior inconclusive consultations. The Love and Hildreth tests are simple, highly specific bedside diagnostic tools that are underutilized in non-specialist clinical practice and should be applied routinely in this clinical setting. Plain radiography should be obtained as a first-line investigation to exclude bony pathology, including cortical erosion and alternative diagnoses such as enchondroma and osteoid osteoma, even though it is typically unremarkable in glomus tumors. MRI is the imaging investigation of choice and enables precise preoperative lesion localization, accurate size assessment, and surgical planning. Histopathological confirmation, including immunohistochemical profiling with SMA and CD34, is essential to establish the definitive diagnosis and subtype. Complete excision via a transungual nail-preserving approach is safe, effective, and associated with excellent long-term functional and cosmetic outcomes, with low recurrence rates when clear margins are achieved. Replacement of the native nail plate as a biological dressing is recommended to optimize cosmetic nail regrowth and minimize postoperative hypersensitivity. Early and accurate diagnosis is paramount to preventing the prolonged and avoidable patient suffering illustrated in this case. Systematic education of non-specialist clinicians regarding the characteristic clinical triad and the diagnostic utility of the Love and Hildreth tests represents the most effective and immediately actionable strategy for reducing diagnostic delay at the population level.
